# Preventing rare contralateral ureteral injury during robotic-assisted nephroureterectomy: lessons from a surgical complication

**DOI:** 10.1186/s12894-025-01729-3

**Published:** 2025-03-06

**Authors:** Yen Ho, Chung-Howe Lai, Kuan-Ting Lin, Ke-Hsun Lin, Syuan-Hao Syu

**Affiliations:** 1 Department of Urology, Wan Fang Hospital, Taipei Medical University, Taipei, Taiwan; 2 Department of Urology, School of Medicine, College of Medicine, Taipei Medical University, Taipei, Taiwan; 3 Graduate Institute of Clinical Medicine, College of Medicine, Taipei Medical University, Taipei, Taiwan

**Keywords:** Contralateral ureteral injury, Robotic nephroureterectomy, Upper tract urothelial carcinoma

## Abstract

Robotic-assisted laparoscopic nephroureterectomy (RALN) is a common approach for upper tract urothelial carcinoma (UTUC), offering advantages such as reduced morbidity and improved recovery. However, contralateral ureteral injury during bladder cuff excision is extremely rare and can be challenging to manage. We present the case of a 56-year-old male with low-grade urothelial carcinoma of the right renal pelvis who underwent RALN. Anatomical distortion during surgery led to misplacement of a surgical clip at the contralateral ureterovesical junction, resulting in obstruction and anuria. Urgent cystoscopy and ureteroneocystostomy with stent placement successfully restored ureteral function. This case highlights the importance of thorough preoperative planning, careful anatomical verification, and heightened intraoperative vigilance to prevent such rare complications in robotic surgery. Ensuring accurate identification of anatomical structures is crucial to avoid inadvertent injury, specifically in complex urologic oncology procedures.

## Introduction

Upper tract urothelial carcinoma (UTUC) is a rare but aggressive malignancy that accounts for approximately 5–10% of all urothelial cancers globally. UTUC primarily affects the renal pelvis and ureter, posing significant challenges in diagnosis and treatment because of its often subtle presentation and complex anatomical involvement [1]. The rarity of the disease, combined with its aggressive nature, makes its management particularly demanding, requiring a balance between oncologic control and preservation of renal function.

The standard treatment for localised UTUC often involves radical nephroureterectomy (RNU) with bladder cuff excision to achieve complete oncologic control and minimise the risk of recurrence [1]. However, RNU is associated with considerable morbidity, especially in patients with preexisting renal impairment. In recent years, robotic-assisted laparoscopic nephroureterectomy (RALN) has emerged as a favourable option because of its minimally invasive nature, offering several advantages over open techniques, such as reduced postoperative pain, shorter hospital stays, and faster recovery [2]. The precision and enhanced visualization provided by robotic systems have revolutionised the surgical approach to UTUC, making complex dissections more feasible and less traumatic for patients.

Despite these advancements, the adoption of robotic technology in urologic oncology presents unique challenges. One of the most significant challenges is the absence of tactile feedback, which forces surgeons to rely heavily on visual cues, potentially increasing the risk of intraoperative complications, such as anatomical misidentifications or inadvertent injuries. A rare but noteworthy complication is injury to the contralateral ureter during bladder cuff excision—a situation that underscores the delicate nature of these procedures and the critical importance of anatomical precision [3].

In this report, we present a unique case of contralateral ureteral injury during RALN, analysing its implications, management strategies, and preventive measures. This case highlights the importance of careful intraoperative planning, advanced visualization techniques, and vigilance in preventing complications, ultimately contributing to improved surgical outcomes in the management of UTUC.

## Case presentation

**Fig. 1:**
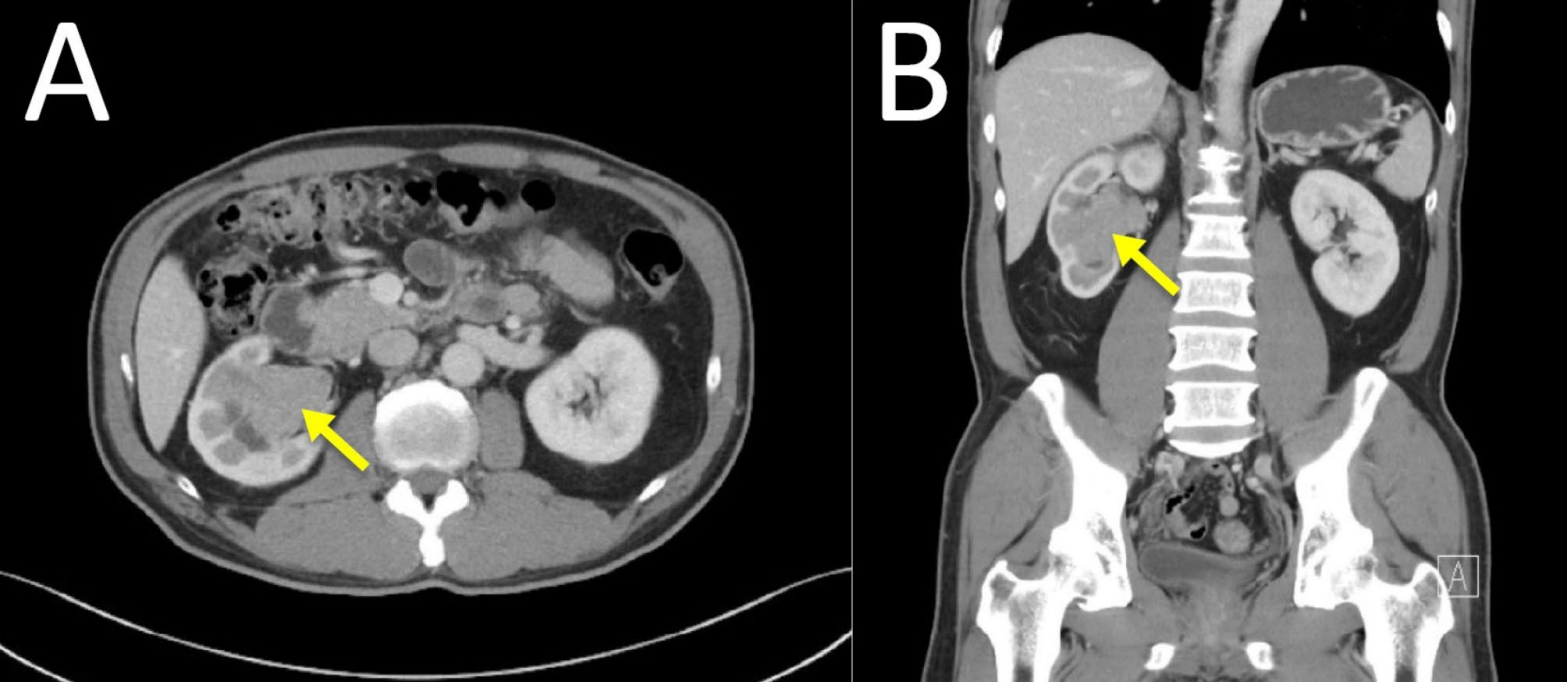
**A**,** B**: Axial and coronal views of the abdominal and pelvic computed tomography (CT) images demonstrate a preoperative enhancing tumoral lesion (arrow) measuring up to 5.75 cm in the right renal pelvis. The lesion involved the renal parenchyma and proximal ureter, with associated right hydronephrosis, indicating obstruction at this site

A 56-year-old male with systemic medical illness presented with persistent right lower quadrant pain and a sensation of urinary fullness. Imaging revealed a renal pelvic mass with hydronephrosis (Fig. 1A, B), and a subsequent biopsy confirmed low-grade, noninvasive papillary urothelial carcinoma. The patient had no history of pelvic surgeries, such as radical prostatectomy, which could complicate surgical anatomy or outcomes. After consultation with a multidisciplinary oncology team, the patient chose to undergo robotic-assisted laparoscopic right nephroureterectomy with bladder cuff excision.

**Fig. 2:**
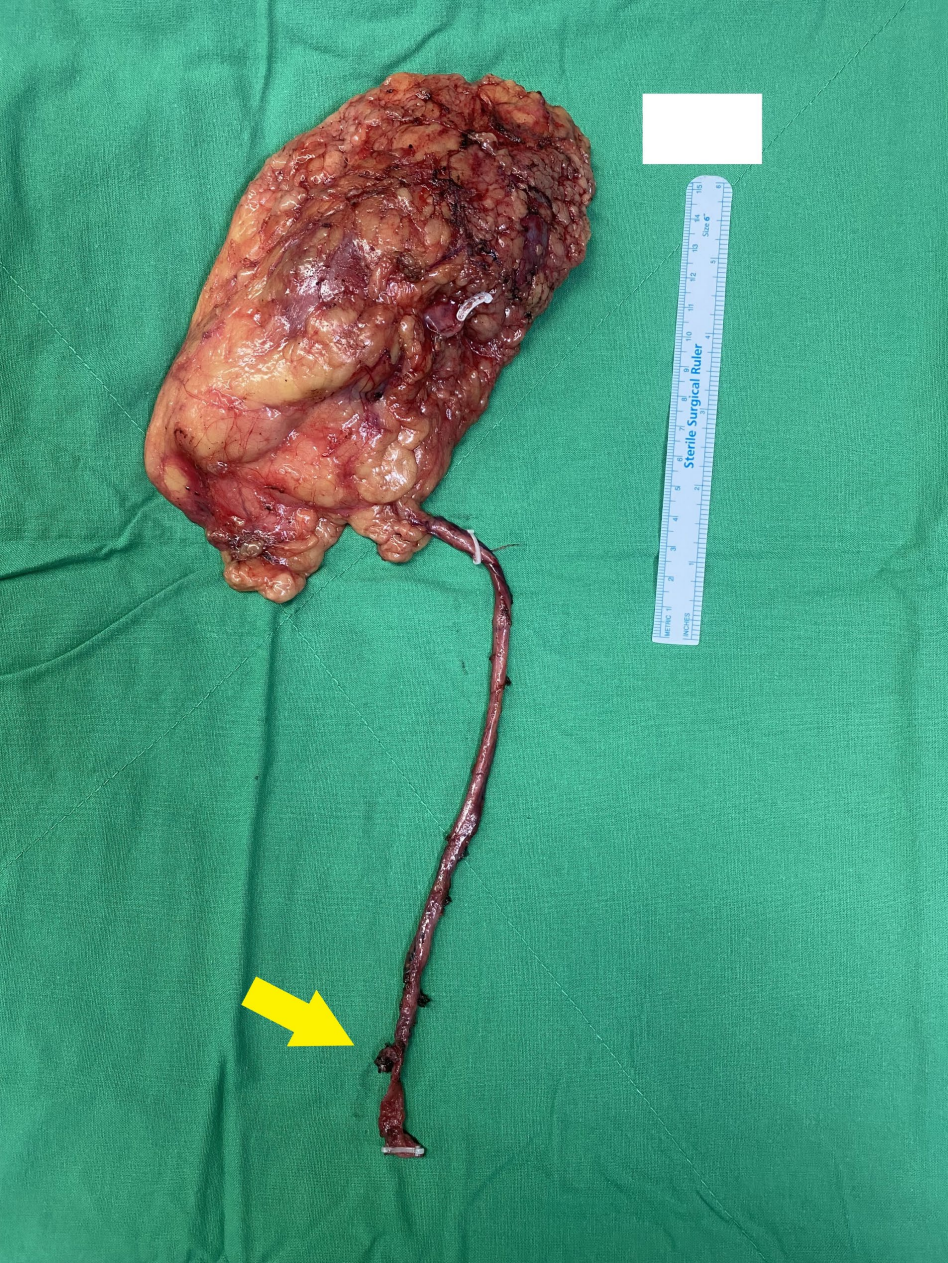
Gross appearance of the right kidney and ureter showing the excised specimen. The kidney has a smooth external surface with visible hilar structures, whereas the ureter has a distinct tubular structure extending distally. The arrow highlights the missing segment of the ureter at its distal end, where the characteristic tubular structure is absent. This finding indicates that the muscle layer was dissected from the posterior bladder wall and extended to the contralateral ureterovesical junction, suggesting inadvertent involvement of the contralateral ureter during the surgical procedure

**Fig. 3:**
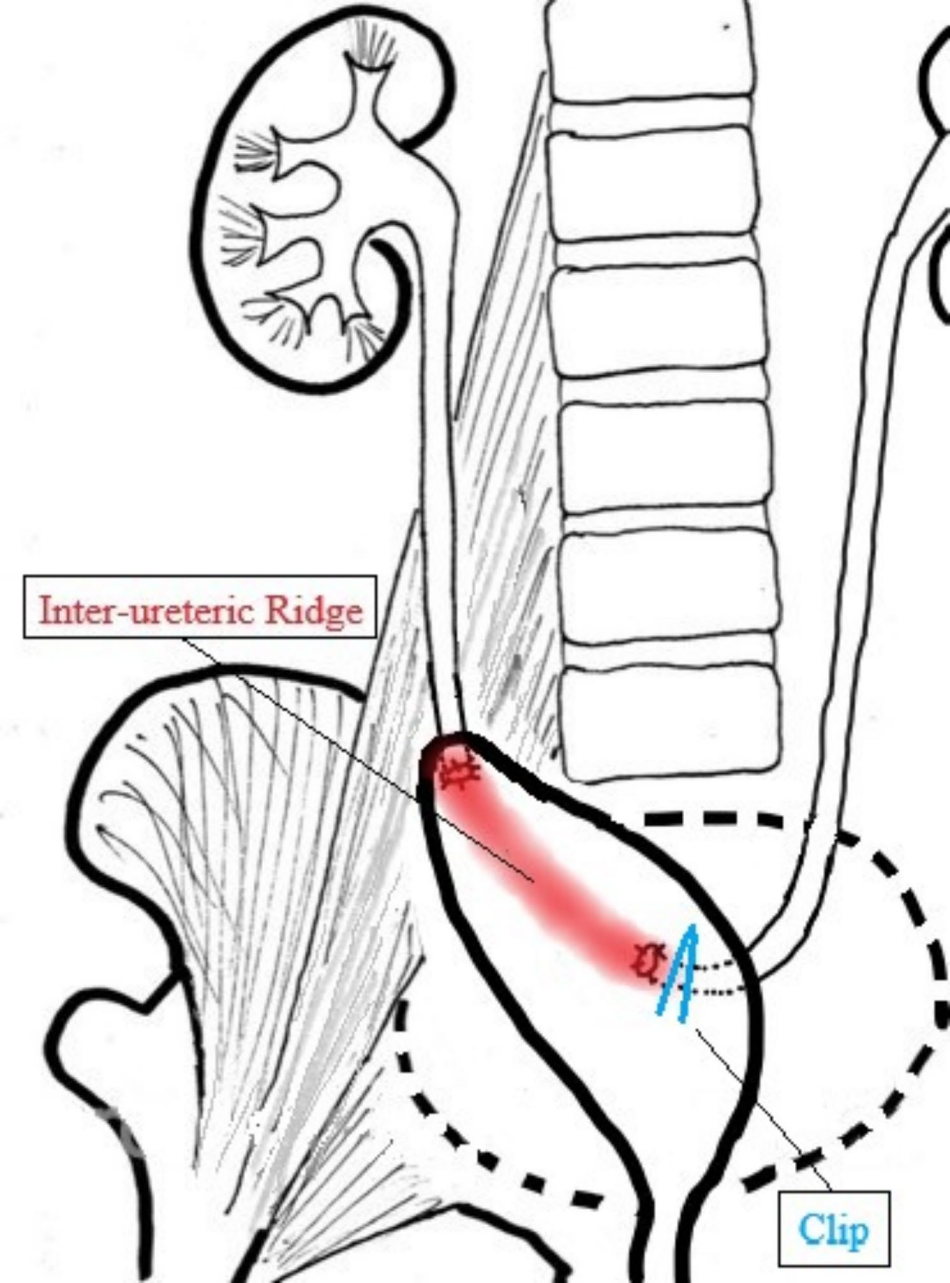
Schematic diagram illustrating the mechanism of contralateral ureteral injury during robotic-assisted nephroureterectomy. The decompressed and deformed bladder, caused by upwards traction of the right ureter, resulted in the left ureterovesical junction shifting towards the right side along with the interureteric ridge structure. During the dissection of the right lower third ureter, the serosal mucosa of the posterior bladder wall was inadvertently dissected, leading to the left ureterovesical junction. A bulging tubular structure, presumed to be the right ureterovesical junction, was identified but was, in fact, the left ureterovesical junction, where the surgical clip was mistakenly applied. The dashed line represents the original contour of the bladder prior to its deformation during the procedure

The procedure began smoothly, with the robotic system providing enhanced three-dimensional visualization, enabling precise dissection of the right renal unit, including the kidney, proximal ureter, and associated structures. As the surgery progressed to the bladder cuff excision phase, the bladder was mobilised into the abdominal cavity to provide better access to the surgical site. This mobilization, however, caused notable anatomical distortion, altering the usual spatial relationships of key structures in the pelvic region. During the excision, the surgical team identified the tubular structure of the right bladder cuff. To secure this structure for resection, a surgical clip was applied to ensure haemostasis and prevent urine spillage (Fig. 2). Unfortunately, owing to the altered anatomy caused by repositioning of the bladder, the tubular structure identified was actually the contralateral ureter. This misidentification and subsequent clipping resulted in obstruction of the left ureter at the ureterovesical junction, leading to postoperative complications. (Fig. 3)

**Fig. 4:**
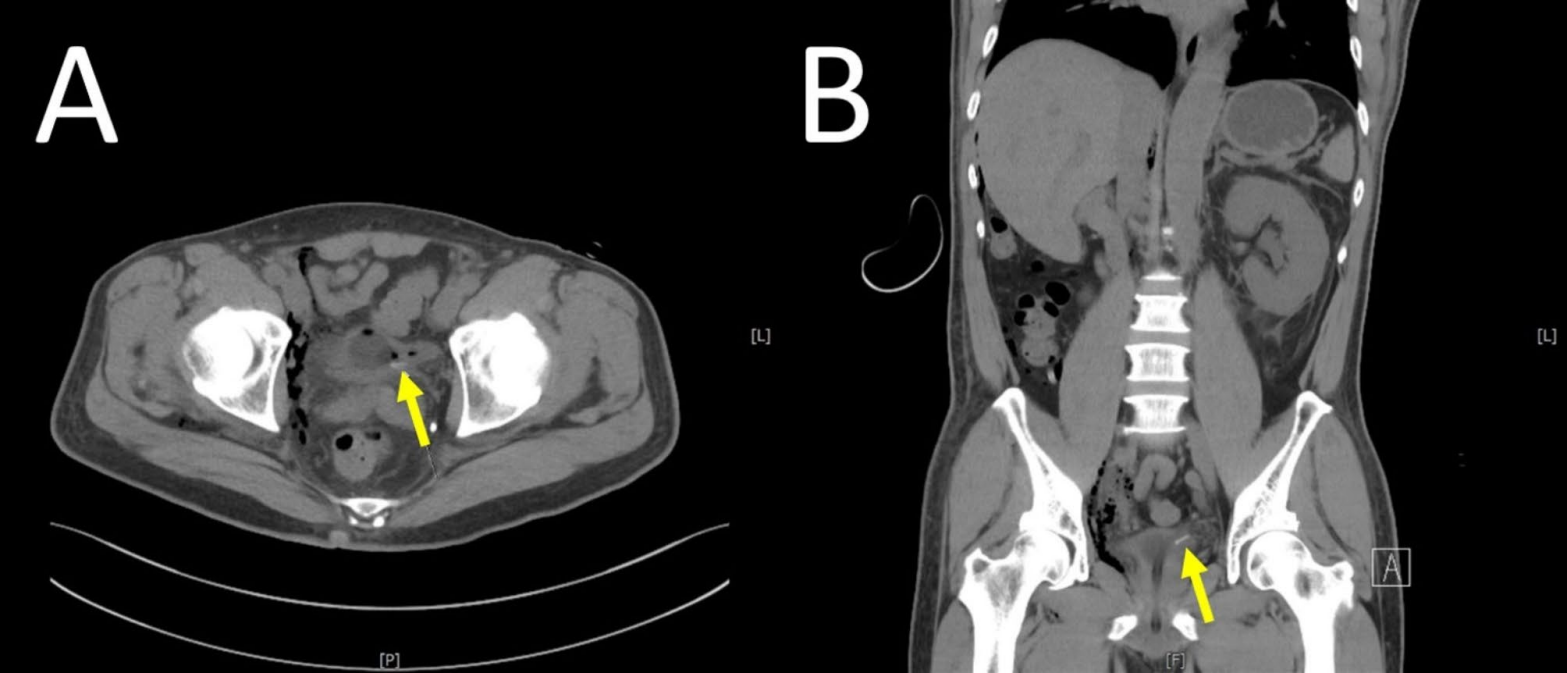
**A**,** B**: Axial and coronal views of the abdominal and pelvic computed tomography (CT) images demonstrate a linear hyperdense structure at the left ureterovesical junction. The lesion (arrow) is consistent with the appearance of a possible Hem-o-lok surgical clip, suggesting obstruction at this site. Changes in the surrounding soft tissue indicated mild secondary hydronephrosis of the left kidney

**Fig. 5:**
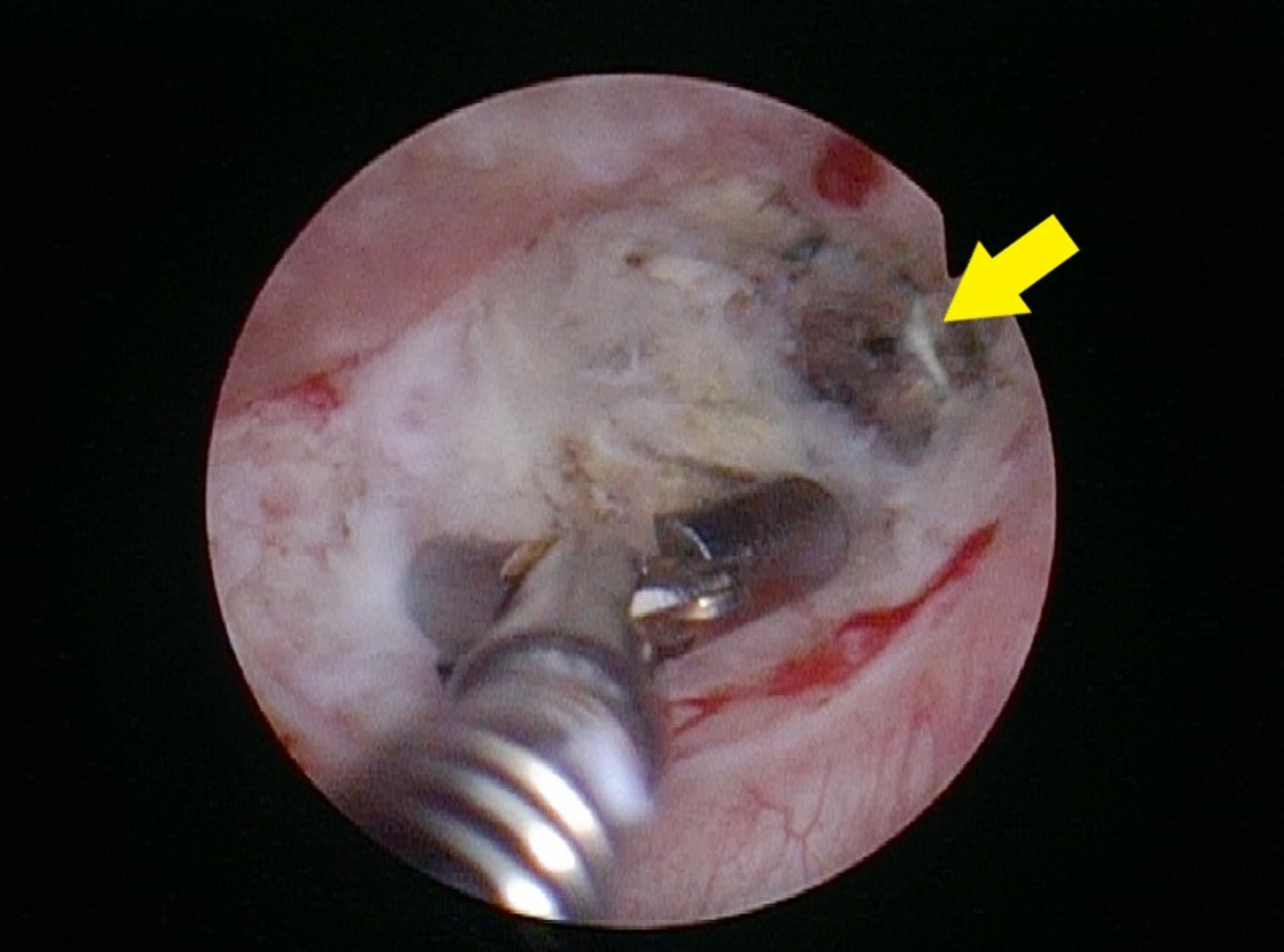
Immediate cystoscopic view demonstrating significant mucosal laceration and a small fissure extending towards the abdominal cavity (not visible in this image). The arrow highlights the misplaced surgical clip at the left ureterovesical junction, which resulted in ureteral obstruction and necessitated urgent surgical management

**Fig. 6:**
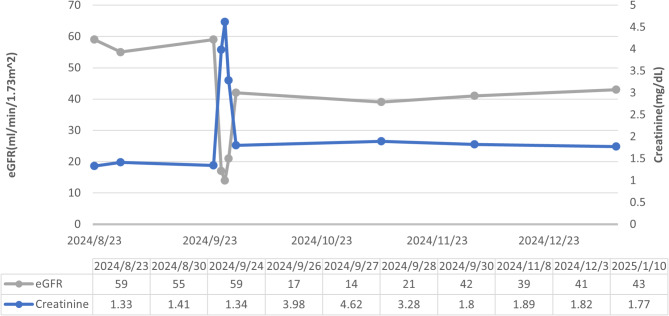
Trends in renal function over time, showing the serum creatinine level (mg/dL) and estimated glomerular filtration rate (eGFR, mL/min/1.73 m²). A significant decline in renal function occurred on September 27, 2024 (creatinine, 4.62 mg/dL; eGFR, 14 mL/min/1.73 m²), followed by gradual recovery after intervention. By January 10, 2025, renal function had stabilised, reflecting the effectiveness of management

Postoperatively, the patient developed anuria within hours. He also complained of abdominal dullness and mild tenderness. Renal ultrasonography revealed left-sided hydronephrosis, and computed tomography confirmed an obstruction at the left vesicoureteral junction caused by the surgical clip (Fig. 4A, B). Emergency cystoscopy (Fig. 5) and left ureteroneocystostomy with double-J stent placement were performed, successfully restoring ureteral continuity. Following the intervention, the patient’s renal function steadily improved, and he was discharged three days later with plans for continued oncological and functional follow-up (Fig. 6).

During the follow-up, the patient denied experiencing physical discomfort. Renal ultrasonography revealed complete resolution of left-sided hydronephrosis, indicating restored renal drainage and functional recovery. The double-J stent was subsequently removed without complications. Ureteroscopy revealed only mild stenosis at the site of ureteroneocystostomy, with no evidence of significant obstruction or stricture. Cystoscopic examination of the bladder wall demonstrated intact mucosal healing, confirming the success of the surgical repair. These findings were reassuring, supporting a positive prognosis and the effectiveness of the intervention. The patient was scheduled for ongoing monitoring to ensure long-term renal function preservation and to detect any signs of recurrence or further complications.

## Timeline

July 2024: The patient presented with persistent right lower quadrant pain and fullness. Imaging revealed a renal pelvic mass with hydronephrosis.

August 2024: Biopsy confirmed low-grade, noninvasive papillary urothelial carcinoma. A multidisciplinary oncology team recommended robotic-assisted laparoscopic nephroureterectomy.

September 25th, 2024: Surgery was performed, and during the bladder cuff excision phase, a surgical clip was mistakenly applied to the contralateral ureter due to anatomical distortion.

Postoperative Day 1: The patient developed anuria. Imaging revealed left-sided hydronephrosis and obstruction at the left ureterovesical junction caused by the misplaced clip. Emergency cystoscopy and left ureteroneocystostomy with double-J stent placement were performed, restoring ureteral continuity.

October 4^th,^ 2024: The patient was discharged with improved renal function and arrangements for ongoing follow-up.

December 2024: Follow-up renal sonography revealed no hydronephrosis of the left kidney. Double J stent removal was performed. Only mild stenosis of the left ureter was noted via ureteroscopy, and mucosal healing of the bladder wall seemed to be intact via cystoscopy.

## Discussion

### Incidence and mechanisms of ureteral injury

Ureteral injuries during robotic-assisted urologic procedures are infrequent but can lead to severe consequences. The incidence of ureteral injuries during nephroureterectomy is generally low, with most involving the ipsilateral ureter or bladder cuff area [1]. Contralateral ureteral injury, as observed in this case, is exceedingly rare, with few reports in the literature. Factors contributing to such injuries include anatomical distortion during surgery, procedural complexity, and an overreliance on visual cues in the absence of haptic feedback [1, 4].

### Challenges of robotic surgery in urology

Robotic-assisted surgery offers significant benefits, including superior visualization, precision, and reduced morbidity; however, it also has inherent limitations. One of the primary challenges is the lack of tactile feedback, which necessitates a heavy reliance on visual cues to differentiate anatomical structures [4]. This can be particularly problematic in surgeries involving anatomical mobilization, as illustrated in this case, where bladder mobilization led to significant landmark distortion. Intraoperative fluorescence imaging techniques, such as the use of indocyanine green (ICG), have been suggested as potential solutions to enhance visualization and prevent ureteral injuries [4, 5].

In addition to the absence of tactile feedback, the steep learning curve associated with robotic surgery can contribute to the risk of complications. While robotic platforms offer enhanced dexterity and precision, attaining proficiency requires significant practice, and even experienced surgeons may face challenges in complex cases [5]. The importance of specialised training and familiarity with robotic systems cannot be overstated, as it plays a crucial role in minimising intraoperative errors [6].

### Risk factors for contralateral ureteral injury

1. Anatomical Variations and Distortions: Mobilization of the bladder into the abdominal cavity can distort anatomical landmarks, making it challenging to differentiate the ureters. This is particularly concerning when dealing with the contralateral side, as misidentification can lead to inadvertent clipping [5]. Moreover, anatomical variations such as duplex ureters or aberrant vascular structures may increase the risk of misidentification [7].
2. Surgical Complexity: The complexity of bladder cuff excision during nephroureterectomy involves balancing oncologic control while avoiding injury to adjacent structures. This balance can be particularly challenging without adequate identification of anatomical landmarks [1]. The risk is further heightened in patients with previous abdominal or pelvic surgeries, where scar tissue and adhesions can obscure normal anatomy, complicating dissection and increasing the risk of injury [8].
3. Absence of Tactile Feedback: The lack of tactile sensation during robotic surgery necessitates reliance on visual interpretation, which can be misleading. In cases involving significant anatomical mobilization, this limitation increases the risk of misidentification [4]. Additionally, the absence of haptic feedback can make it difficult to assess tissue tension, increasing the likelihood of inadvertent clamping or injury to delicate structures such as ureters [8].

### Management of iatrogenic ureteral injuries

The successful management of ureteral injuries hinges on prompt recognition and intervention. In this case, immediate imaging and subsequent surgical repair prevented permanent renal damage. Ureteroneocystostomy is a well-established approach for managing distal ureteral injuries and involves reimplantation of the ureter into the bladder with a double-J stent to ensure patency and minimise the risk of stricture formation [2, 3]. Early postoperative monitoring and prompt recognition of symptoms such as anuria are essential for preventing long-term sequelae of ureteral injuries [1].

In instances of extensive ureteral damage, other reconstructive options may be considered. Boari flap ureteral reimplantation or psoas hitch procedures can be employed to bridge larger defects in the distal ureter, providing additional flexibility in managing complex injuries [8]. In some cases, autotransplantation of the kidney may be necessary when ureteral continuity cannot be restored using conventional methods [8].

In addition to immediate surgical repair, the timing of ureteral reconstruction remains a critical consideration in the management of iatrogenic ureteral injuries. While immediate repair is often feasible, delayed or “cold” reconstruction may be preferred in cases where significant inflammation, infection, or tissue oedema is present, as these factors can compromise surgical outcomes. A delayed reconstruction period allows the local inflammatory response to subside, potentially reducing the risk of postoperative stricture or anastomotic failure [9]. 

As a temporising measure, nephrostomy placement can provide effective urinary diversion and renal preservation, allowing for stabilization before definitive surgical repair. This approach is particularly useful in haemodynamically unstable patients or those with extensive tissue damage [10]. Ultimately, the decision between immediate and delayed reconstruction should be tailored to the individual patient’s clinical condition, considering the extent of injury, the presence of infection, and overall renal function. Multidisciplinary collaboration is essential to optimise outcomes in these complex cases [11]. 

## Preventive strategies

### Preoperative planning

Preoperative imaging, such as contrast-enhanced CT or MRI, can delineate the anatomy and identify anatomical variations that may increase the risk of ureteral injury. In high-risk cases, the placement of ureteral stents before surgery serves as an additional safeguard by making the ureters more identifiable intraoperatively [3, 4]. Additionally, three-dimensional reconstruction of imaging data has been shown to enhance preoperative planning by providing a more comprehensive view of the patient’s anatomy, thereby reducing intraoperative surprises and assisting in the identification of critical structures [7].

### Intraoperative techniques

1. Fluorescence-Guided Imaging: The use of ICG fluorescence during robotic-assisted surgery provides real-time visualization of the ureters, enhancing their ability to avoid accidental injury [4]. Advances in near-infrared fluorescence imaging have further improved the accuracy of ureteral identification, making it a valuable tool in complex robotic surgeries [7].
2. Sequential Dissection: Performing layered dissection during bladder cuff excision facilitates more precise identification and protection of the ureters [5]. This approach ensures systematic progression through tissue planes, reducing the risk of inadvertently injuring nontarget structures.
3. Routine Intraoperative Cystoscopy: Intraoperative cystoscopy is a valuable adjunct for confirming ureteral integrity during critical steps, such as bladder cuff excision. It allows direct visualization of ureteral orifices, ensuring that no unintentional ligation or injury has occurred [3].

### Postoperative vigilance

Early postoperative symptoms, such as anuria or significant flank pain, should prompt immediate imaging to assess ureteral integrity. Rapid diagnosis and timely intervention are crucial for preventing permanent renal damage [2, 12]. Postoperative monitoring protocols should include routine renal ultrasound and serum creatinine measurements to ensure early detection of any signs of obstruction or renal impairment [7].

### Strengths and limitations

This case report provides valuable insights into a rare and significant complication of robotic-assisted nephroureterectomy, offering a detailed account of its diagnosis, management, and outcomes. The strength of this report lies in its emphasis on the importance of intraoperative vigilance and anatomical verification, which are critical for preventing contralateral ureteral injury. The inclusion of postoperative imaging, ureteroscopy, and patient-assessed outcomes highlights the thorough approach taken to ensure recovery and demonstrates the effectiveness of the surgical intervention.

However, there are limitations to this case report. As a single case, the findings lack generalizability to broader clinical contexts, as patient anatomy, surgical techniques, and robotic systems may vary significantly. Additionally, the absence of long-term follow-up data limits the understanding of potential delayed complications, such as recurrent stenosis or impaired renal function. Furthermore, reliance on subjective patient-reported outcomes introduces a degree of bias in evaluating recovery. Future studies with larger cohorts and extended follow-up are needed to validate the lessons learned and refine preventive strategies for this rare complication.

## Conclusion

This case highlights a rare yet avoidable complication of robotic-assisted nephroureterectomy—contralateral ureteral injury. This underscores the importance of meticulous preoperative planning, careful intraoperative identification of anatomical structures, and the adoption of advanced technologies such as fluorescence imaging. As the use of robotic systems continues to expand in urologic oncology, surgeons must remain vigilant regarding the limitations of these technologies and adopt preventive strategies to mitigate associated risks. Integrating these lessons into clinical practice will help enhance the safety and efficacy of robotic-assisted urologic surgeries, ultimately leading to better outcomes for patients with complex oncologic conditions.

## Data Availability

The datasets generated and/or analyzed during the current study are not publicly available due to patient confidentiality restrictions. However, they are available from the corresponding author upon reasonable request, provided the request complies with institutional and ethical guidelines.
